# Supplementary figures and images for: B6 Mouse Strain: The Best Fit for LPS-Induced Interstitial Cystitis Model
Source: Int J Mol Sci. 2021 Nov 8;22(21):12053. doi: 10.3390/ijms222112053 (PMC8585067; doi:10.3390/ijms222112053)

Supplementary Figure S1: The change of body weight in each group at different time point.

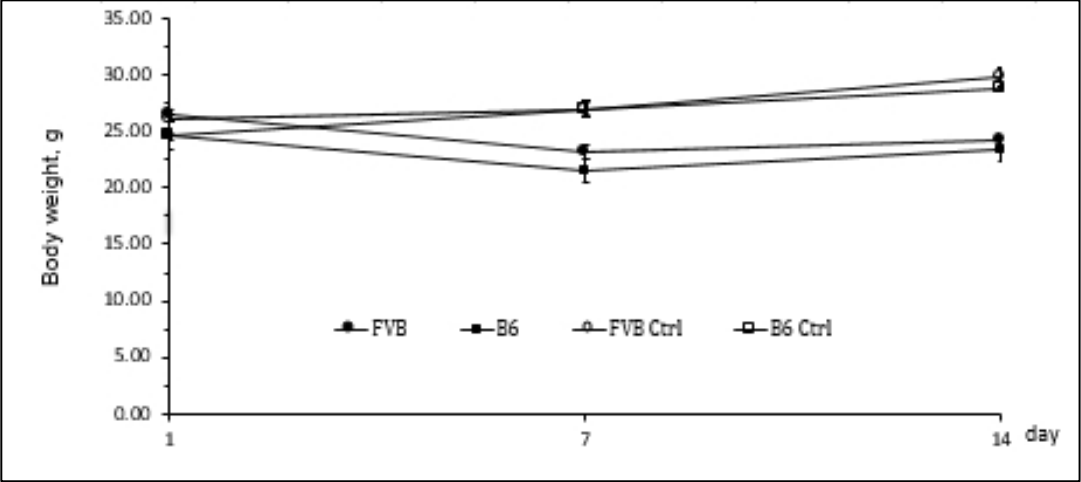

Supplement: Supplementary file 1 [file ijms-22-12053-s001.zip › ijms-1433296-supplementary.pdf]
